# Validation of the Portuguese Version of the Kansas City Cardiomyopathy Questionnaire-12

**DOI:** 10.3390/jcdd10040162

**Published:** 2023-04-07

**Authors:** Mariane Cecilia dos Reis, Juliana Araújo Nascimento, Geisa Nascimento de Andrade, Ana Cláudia de Souza Costa, Julio Yoshio Takada, Antonio de Padua Mansur, Edimar Alcides Bocchi, Gianni Mara Silva dos Santos, John A. Spertus, Naomi Kondo Nakagawa

**Affiliations:** 1 Education, Assessment and Intervention in Cardiopulmonary Group, Faculdade de Medicina da Universidade de São Paulo, Sao Paulo 01246-903, SP, Brazil; 2 Department of Nursing, Pontifical Catholic University of Minas Gerais, Poços de Caldas 37714-620, MG, Brazil; 3 Unidade Clínica de Coronariopatias Crônicas, Instituto do Coração (InCor), Hospital das Clinicas HCFMUSP, Faculdade de Medicina, Universidade de SaoPaulo, Sao Paulo 05508-030, SP, Brazil; 4 Serviço de Prevenção, Cardiopatia na Mulher e Reabilitação Cardiovascular, Instituto do Coração (InCor), Hospital das Clinicas HCFMUSP, Faculdade de Medicina, Universidade de São Paulo, Sao Paulo 05508-030, SP, Brazil; 5 Unidade Clínica de Insuficiência Cardíaca, Instituto do Coração (InCor), Hospital das Clinicas HCFMUSP, Faculdade de Medicina, Universidade de São Paulo, Sao Paulo 05508-030, SP, Brazil; 6 Pró-Reitoria de Pós-Graduação e Pesquisa, Universidade Federal de São Paulo, Sao Paulo 04021-001, SP, Brazil; 7 Healthcare Institute for Innovations in Quality, Saint Luke’s Mid America Heart Institute, University of Missouri, Kansas City, MO 64110, USA

**Keywords:** Kansas City Cardiomyopathy Questionnaire, heart failure, quality-of-life, psychometric properties

## Abstract

The Kansas City Cardiomyopathy Questionnaire-12 (KCCQ-12) is a simple, feasible, and sensitive questionnaire developed in English for assessing the health status (symptoms, function, and quality of life) of patients with heart failure (HF). We aimed to assess the internal consistency and construct validity of the Portuguese version of KCCQ-12. We administered the KCCQ-12, the Minnesota Living Heart Failure (MLHFQ), and the New York Heart Association (NYHA) classification by telephone. Internal consistency was assessed with Cronbach’s Alpha (α-Cronbach) and construct validity with correlations to the MLHFQ and NYHA. Internal consistency was high (α-Cronbach = 0.92 for the Overall Summary score and 0.77–0.85 for the subdomains). Construct validity was supported by finding high correlations between the KCCQ-12 Physical Limitation and the Symptom Frequency domains with the physical domain of the MLHFQ (r = −0.70 and r = −0.76, *p* < 0.001 for both) and the Overall Summary scale with NYHA classifications (r = −0.72, *p* < 0.001). The Portuguese version of KCCQ-12 has high internal consistency and shows a convergent construct validity with other measures quantifying the health status of patients with chronic HF and can be used confidently in Brazil for research and clinical care.

## 1. Introduction

Heart failure (HF) is a global health challenge with high morbidity and mortality that affects approximately 64.3 million adults worldwide [1,2]. Moreover, the prevalence of HF is increasing, particularly with aging, and a patient’s quality of life is negatively affected by progressive symptoms and functional capacity reduction [3]. The Minnesota Living Heart Failure Questionnaire (MLHFQ) and the Kansas City Cardiomyopathy Questionnaire (KCCQ) are the most widely used tools for assessing the health status (symptoms, function, and quality of life) of patients with HF [4,5,6,7,8,9].

A shorter, 12-item version of the KCCQ (KCCQ-12) was created to preserve the excellent psychometric properties of the original KCCQ 23-item version [5] and has been endorsed by the International Consortium of Health Outcomes Measurement to quantify the value of heart failure care [10]. The KCCQ-12, beyond being an easy-to-perform, sensitive tool for eliciting patients’ health status and clinical changes over time, has strong prognostic importance for hospitalizations and all-cause mortality in patients with HF [5,9,11]. Accordingly, the KCCQ-12 is being increasingly used in clinical practice [9]. For practitioners to adopt the KCCQ-12 to directly measure their patients’ health status, empirical evidence supporting the psychometric properties of the KCCQ-12 translations is needed, even if the state-of-the-art translation processes are followed [12].

The Portuguese version of KCCQ-12 could be widely adopted to benefit the clinical management and health care of approximately 292 million individuals from nine Portuguese-speaking countries. To support its use in Brazil, this study aimed to assess its internal consistency and construct validity in Brazilian patients with chronic HF by testing its convergence with the MLHFQ and clinician-assigned New York Heart Association (NYHA) classification, which are commonly used to characterize the functional status of patients with HF [13].

## 2. Materials and Methods

The Ethical Committee of Heart Institute of Clinics Hospital (InCor-HCFMUSP), University of São Paulo Medical School, approved this cross-sectional study (CAAE 74659717.7.0000.0065). This work followed the ethical guidelines of the Helsinki Declaration. We included stable HF patients with reduced left ventricular ejection fraction (LVEF ≤ 40%), both sexes, and aged ≥ 18 years from the Heart Failure Outpatient Clinic (October 2020 to December 2021). We excluded patients with cognitive and/or neuromotor impairment, patients with chronic restrictive or obstructive pulmonary disease, and cardiac or respiratory infection in the last 90 days. Electronic records were reviewed to abstract participants’ demographic and clinical data. One of the two investigators contacted each patient. We randomly administered the KCCQ-12, MLHFQ, and NYHA classification by telephone, due to the COVID-19 pandemic and recorded the interviews.

### 2.1. KCCQ-12, MLHFQ, and NYHA Functional Class

Briefly, the KCCQ-12 evaluates the patient´s health status over the last two weeks. The KCCQ-12 is composed of 4 domains: (1) physical limitation (items 1a, 1b, 1c), (2) symptom, frequency (items 2, 3, 4, 5), quality-of-life (items 6, 7), and social limitation (items 8a, 8b, 8c), [5] which are averaged to generate the Overall Summary score. Scores range from 0 to 100, with higher scores indicating fewer symptoms or limitations and better quality of life. To support its interpretation, cross-sectional scores of 0–24 indicate very poor to poor health status, 25–49 poor to fair, 50–74 fair to good, and 75–100 good to excellent health status [5,6,14]. Changes of 5, 10, and ≥20 points indicate small but clinically important, moderate to large, and large to very large changes in patients’ health status. 

The MLHFQ assesses the patient´s health status over the last month. The MLHFQ score ranges from zero to 105 units (the worst possible health status). MLHFQ is composed of 21 items divided into (1) physical domain (items 1–7, 12, 13) and (2) emotional domain (items 17–21). The remaining items (8, 9, 10, 11, 14, 15, and 16) also compose the overall score [4,13,15]. The overall MLHFQ score can also be divided into poor and fair (≥53 units) and good and excellent health status (<53 units) [6].

The NYHA classification was performed with the aid of the Specific Activity Scale [16], designed as a self-administered method for categorizing health status into four functional classes: I (the best) to IV (the worst functional status) [17].

### 2.2. Statistical Analysis

The sample size calculation to ensure robust internal consistency and construct validity was based on the Consensus-Based Standards for selecting Health Measurement Instruments [18]. Each item of the KCCQ-12 required 5 to 10 individuals [18,19], resulting in 120 patients.

Demographic and clinical characteristics of the patients were summarized as numbers and percentages for categorical variables or expressed as means and standard deviation for continuous variables. Internal consistency of the Portuguese version of KCCQ-12 was assessed with Cronbach´s Alpha (α-Cronbach). The α-Cronbach ranges between 0 and 1 (1, the highest “cohesiveness” of different items and higher correlation between them within each domain) [20].

We used the Spearman Coefficient Correlation to assess the convergence of construct validity between the KCCQ-12, MLHFQ, and NYHA functional classification. We also analyzed the construct validity between specific domains of KCCQ-12 (physical limitation and symptom frequency domains) and the MLHFQ physical domain.

Additionally, we performed the Fisher Test to examine the correlation between KCCQ-12 (<50 as poor/fair vs. 50 as good/excellent), MLHFQ (<53 as good/excellent vs. 53 as poor/fair) [3], and NYHA classification. Finally, we categorized patients into two sub-groups by the severity of their NYHA class (asymptomatic/mild vs. moderate/severe status) [17].

A *p*-value < 0.05 was considered statistically significant, and no adjustments for multiple comparisons were made, due to the high correlation of the constructs being compared. The Statistical Package for the Social Science Software, version 24 (SPSS, International Business Machines Corp., Chicago, IL, USA), was used to test internal consistency, and Statistical Analysis System, University Edition^®^ (SAS Institute Inc., Cary, NC, USA), was used for construct validity.

## 3. Results

Our study included a total of 124 patients. Most patients were male and hypertensive, had moderate functional incapacity (NYHA III), and had non-ischemic etiology (Table 1).

The internal consistency of the overall score of the Portuguese version of KCCQ-12 and each of the four domains were high and are shown in Table 2.

The construct validity of KCCQ-12 and MLHFQ showed convergence in the overall score (r = −0.77, *p* < 0.001). The correlation between the KCCQ-12 Physical Limitation and Symptom Frequency domains and the physical domain of the MLHFQ (r = −0.70 and r = −0.76, respectively, *p* < 0.001 for both) were also high. The NYHA classification was also highly correlated with the KCCQ-12 Overall Summary score (r = −0.72, *p* < 0.001) but less so with the MLHFQ overall score (r = 0.59, *p* < 0.001). The distribution of patients in each sub-group was significantly different when scores of KCCQ-12, MLHFQ, and NYHA were dichotomized (Table 3).

## 4. Discussion

Confirming the psychometric properties of a patient-reported outcome in countries where it was not developed can increase confidence in its use for research and clinical practice. To address this need for Brazil, we independently tested the internal consistency of the Portuguese version of KCCQ-12 and compared its construct validity with MLHFQ and NYHA to quantify health status in HF patients with reduced LVEF. We found high internal consistency between items within each KCCQ-12 domain and its Overall Summary score. Additionally, we observed high construct validity between KCCQ-12 and other measures of patients’ health status, including the MLHFQ and NYHA. These results support the use of the Portuguese version of KCCQ-12 in Brazil.

Psychometric properties of self-report questionnaires should be tested in several scenarios with different populations, cultures, and languages to guarantee the safety of the questionnaires [12,21]. MLHFQ and KCCQ-23 are commonly used to assess health status in the clinical care of HF patients and research, mainly because they have good reliability (test-retest analysis) and responsiveness to clinical changes [4,19]. The Evaluating Patient-Reported Outcomes (EMPRO) is a standardized tool to assess agreement between scales (the worst agreement: 0 to 100 points, the most robust agreement) to evaluate relevant attributes of self-report questionnaires, such as reliability, validity, sensitivity to clinical changes, and interpretability, among others [22]. When using EMPRO, some investigators showed higher performance of KCCQ-23 in comparison with the MLHFQ in interpretability (72 vs. 44 points, respectively) and its sensitivity to clinical changes (94 vs. 67 points, respectively) in patients with HF [22]. The short KCCQ-12 was created to be a more feasible version of the original 23-item version to support routine clinical use, as well as research [5]. Our study showed novel supportive data for using the Portuguese version of KCCQ-12 in patients with HF. We found high internal consistency in domains and the overall KCCQ-12, similar to the English version previously described in men and women [8]. We also showed excellent construct validity between KCCQ-12 with MLHFQ and NYHA by observing strong and convergent trends among them.

KCCQ-12 was recently pointed out as the most reliable tool for evaluating health status, clinical changes, and prognostic outcomes in patients with HF [9,10]. In addition, lower scores of KCCQ-12 (<50 units) are associated with worse NYHA functional capacity [9,11]. Our results are in line with these studies. Among 124 HF patients with reduced LVEF, 50% (n = 62) showed lower scores of KCCQ-12, and 45% (n = 56) showed worse NYHA classification (III and IV). Nineteen subjects (15%) had divergent results between KCCQ-12 and NYHA.

In the literature, investigators showed that differences in KCCQ-12 along clinical follow-up classify clinical changes in minor (5 points), moderate to large (10 points), or large to very large differences (20 points) [14]. Decrements of 10 units in KCCQ-12 are predictive of increased composite risks of cardiovascular hospitalization and death over 30 days [HR: 1.13 (CI: 1.08–1.19)] [12], one year [HR: 1.07 (CI: 1.05–1.09)] [11], and two years [HR: 1.21 (CI: 1.20–1.22)] [9]. On the other hand, increments ≥ 5 units in KCCQ-12 determine risk reduction of composite hospitalization and all-cause mortality in patients with reduced LVEF [HR: 0.73 (CI: 0.59–0.89)] [13]. In this context, co-morbidities, such as diabetes, may increase risk severity of HF and mortality [23], as well as drugs treatment optimization may impact on health status. For instance, clinical trials showed that sacubitril/valsartan are effective to improve health status using KCCQ [24] and to reduce mortality in patients with reduced LVEF [25].

Our work should be interpreted in the context of the following potential limitations. We assessed only two psychometric properties, internal consistency and construct validity. While further exploration of its test-retest reliability and responsiveness are needed, without first establishing its validity and internal reliability, such studies would not be warranted. The COVID-19 pandemic precluded in-person visits, and other assessments of participants’ health status could not be performed. Another aspect is that, while there are several Portuguese-speaking countries, covering 1–2% of the world’s 292 million inhabitants with HF, we focused on Brazil [26]. The 36% female participation could be reported as a sex imbalance. However, it can be commonly observed due to lower prevalence of women with HF and reduced LVEF, despite its greater severity in women than in men [27]. In addition, KCCQ-12 has adequate psychometric properties in male and female with HF [8]. Finally, we validated the Portuguese version of KCCQ-12 in HF patients with reduced LVEF, and future studies should confirm these properties in patients with HF and preserved LVEF. However, all prior studies have found comparable performance and prognostic properties of the KCCQ patients with HF and both reduced and preserved LVEF [9,28].

In the current work, we focused on the internal consistency and construct validity analysis of the Portuguese version of KCCQ-12 in HF patients. This cross-sectional study is the initial step to further validation of its prognostic significance including serial assessments.

## 5. Conclusions

The Portuguese version of KCCQ-12 has excellent internal consistency and construct validity in patients with reduced LVEF and can confidently be used in research and clinical care.

## Figures and Tables

**Table 1:** Demographic and clinical characteristics of outpatients with HF.

	Overall (n = 124)
Age, years (%)	
≤39	7 (6)
40–59	52 (42)
≥60	65 (52)
Men, n (%)	80 (64)
Body mass index, kg/m2	27 ± 5
Left ventricular ejection fraction, %	30 ± 6
New York Heart Association class, n (%)	
I	24 (20)
II	31 (25)
III	66 (53)
IV	3 (2)
Etiology, n (%)	
Ischemic	41 (33)
Non-ischemic	83 (67)
Comorbidities, n (%)	
Hypertension	81 (65)
Diabetes mellitus	47 (38)
Dyslipidemia	35 (28)
Prior myocardial infarction	36 (29)
Atrial fibrilation	29 (23)
Cardiac implantable electronic devices	18 (15)
Chronic kidney disease	12 (10)
Stroke	2 (2)
Medications, n (%)	
Angiotensin-converting enzyme inhibitors	77 (62)
Angiotensin receptor blockers	41 (33)
Statins	82 (66)
Betablockers	122 (98)
Antiarrhythmics	16 (13)
Anticoagulants	29 (23)
Antiplatelets	51 (41)
Digitalis	20 (16)
Nitrates	28 (23)
Diuretics	117 (94)
Other antihypertensive drugs	40 (32)
Smoking history Pack/years	13 ± 26
Smoking classification, n (%)	
Non-smoker	75 (60)
Ex-smoker	43 (35)
Smoker	6 (5)
Level of schooling, n (%)	
Illiterate to education < 4 years	29 (23)
Education ≥ 4 years to Higher Education	85 (68)
Unknown	10 (9)

**Table 2:** Internal consistency of the KCCQ-12.

KCCQ-12	α-Cronbach
Physical limitation	0.77
Symptom frequency	0.79
Quality of life	0.85
Social limitation	0.84
Overall summary	0.92

**Table 3:** Analysis of associations between NYHA, KCCQ-12, and MLHFQ.

		NYHA		
		I–II	III–IV	
Health Status		Asymptomatic/Mild	Moderate/Severe	*p*-Value
**KCCQ-12**				
<50	Poor/Fair	6 (5)	56 (45)	**0.001**
≥50	Good/Excellent	49 (40)	13 (10)	
**MLHFQ**				
<53	Good/Excellent	20 (25)	13 (17)	**0.001**
≥53	Poor/Fair	10 (13)	35 (45)	

## Data Availability

The data presented in this work are available as Supplementary Material (Tables S1 and S2).

## Supplementary Materials

The following supporting information can be downloaded at: https://www.mdpi.com/article/10.3390/jcdd10040162/s1, Table S1: Available Data 1; Table S2: Available Data 2.
