# Supplementary material for: Validation of the Portuguese Version of the Kansas City Cardiomyopathy Questionnaire-12
Source: J Cardiovasc Dev Dis. 2023 Apr 7;10(4):162. doi: 10.3390/jcdd10040162 (PMC10144930; doi:10.3390/jcdd10040162)
Supplement: Supplementary file 1 [file jcdd-10-00162-s001.zip › jcdd-2149304-supplementary.pdf]

Table S1. Available Data 1

hr	nyha	nyha_dic	scholarity	age	lvef	gender	etiol	smok	packyears	height	weight	bmi	hipert	diabete	yslipidemi	atrialfyb	crt	ami	renaldis	stroke	cancer	ACI	ARB	Statins	Other antil	Nitrates	Betablock	Antiarrhytl	Antiplatele	Anticoagul	Digitalis	Diuretics
55770577	2	1	2	62	2	0	2	0	4	1.57	79	2	1	1	1	0	0	0	0	0	0	0	1	1	0	0	1	0	1	0	0	1
55777324	3	2		51	1	1	2	0	105	1.7	134	2	1	0	0	0	0	0	0	0	0	1	0	0	0	0	1	0	0	0	0	1
55481870	3	2	2	50	1	1	2	0	0	1.7	93	2	1	1	1	0	0	0	0	0	0	1	0	1	1	0	1	0	0	0	0	1
55788469	3	2	2	54	1	1	2	0	0	1.63	74	1	0	0	0	0	0	0	0	0	0	1	0	0	0	0	1	0	0	0	0	1
55760743	2	1	2	76	1	1	1	0	55	1.6	100	2	1	0	1	0	0	1	1	0	0	0	1	1	0	0	1	0	1	0	0	1
55790116	3	2	2	46	1	1	2	0	0	1.78	91	1	0	0	0	0	0	0	0	0	0	1	0	0	1	0	1	0	0	0	0	1
55390394	3	2	1	54	1	1	2	0	0	1.73	89	1	1	1	1	0	0	1	1	0	1	1	0	1	1	1	1	0	1	0	0	1
5086459C	3	2	2	63	1	1	2	0	0	1.86	72	1	1	1	1	1	0	0	0	0	0	1	0	1	0	0	1	1	0	1	0	1
55785739	1	1	2	67	1	1	2	0	0	1.72	79	1	0	0	0	0	1	0	0	0	0	1	0	0	0	0	1	0	0	0	0	1
55775622	3	2	1	66	1	1	1	0	42	1.7	86	1	0	0	0	0	0	0	0	0	0	1	0	1	0	0	1	0	1	0	0	1
53742635	1	1	2	54	2	1	2	0	0	1.7	65	1	1	1	0	0	0	0	1	0	0	0	1	1	0	0	1	0	1	0	0	1
55706936	3	2	2	69	1	0	2	0	0	1.55	68	1	0	1	1	0	1	0	0	0	0	0	1	1	0	0	1	0	0	0	1	1
55613740	2	1	2	30	2	0	2	0	10	1.65	73	1	0	0	0	0	0	0	0	0	0	1	0	0	0	0	1	0	0	0	0	1
55770092	2	1	2	40	1	0	2	0	0	1.6	82	2	0	0	0	0	0	0	0	0	0	0	1	0	0	0	1	0	0	0	0	1
5352913K	1	1	2	54	2	0	2	0	0	1.48	55	1	0	1	0	0	1	0	0	0	0	0	1	1	0	0	1	0	0	0	0	1
55788080	1	1	2	47	2	1	2	0	35	1.82	100	2	0	0	0	0	0	0	0	0	0	1	0	0	1	1	1	0	0	0	0	1
2979371I	1	1	2	64	1	1	2	0	0	1.63	65	1	1	0	0	0	0	0	0	0	0	1	0	0	0	0	1	0	0	0	0	1
55603816	3	2		68	1	1	2	0	41	1.88	96	1	1	1	0	0	0	0	0	0	0	0	1	1	0	0	1	0	1	0	0	1
55710514	3	2	2	65	1	0	2	0	0	1.68	80	1	1	0	0	1	2	0	0	0	0	0	1	0	0	0	1	0	0	0	1	1
55789620	2	1	2	64	2	1	2	0	0	1.64	77	1	1	1	0	1	0	0	0	0	0	0	1	1	0	0	1	0	1	0	0	1
77081413	2	1	2	70	1	1	1	0	0	1.85	84	1	1	1	0	0	2	1	1	0	0	1	0	1	1	1	1	1	0	0	0	1
55790461	3	2	1	63	1	0	2	0	0	1.46	54	1	0	0	0	0	0	0	0	0	0	1	0	0	0	0	1	0	1	0	1	1
55613421	3	2	1	56	2	1	2	0	0	1.7	89	2	1	1	0	0	0	0	0	0	0	0	1	1	0	0	1	0	0	0	0	1
55790914	3	2	1	58	2	1	2	0	0	1.68	60	1	0	1	0	0	0	0	0	0	0	1	0	1	0	0	1	0	1	0	0	1
55746283	3	2	2	65	2	0	2	0	0	1.7	70	1	1	1	0	0	0	0	0	0	0	0	1	1	1	1	1	0	0	0	0	1
55776979	2	1	2	54	1	0	2	1	40	1.55	50	1	1	0	1	0	0	0	0	0	0	1	0	1	1	1	1	0	1	0	0	1
55783321	4	2	2	59	1	1	1	0	40	1.73	78	1	1	0	0	0	0	1	0	0	0	1	0	1	1	1	1	0	0	1	0	1
2608935F	1	1	1	51	1	1	2	0	5	1.8	73	1	1	0	0	0	0	0	0	0	0	1	0	0	0	0	1	0	0	0	0	1
55609570	3	2	2	37	1	0	2	0	0	1.68	102	2	0	0	0	0	0	0	0	0	0	0	0	0	1	0	1	1	0	0	0	1
90983721	3	2	2	72	1	1	2	0	0	1.88	76	1	1	1	0	1	0	0	0	0	0	1	0	1	0	0	1	0	0	1	0	1
55786816	3	2	1	76	2	1	2	0	4	1.74	82	1	0	1	1	1	0	0	0	0	0	1	0	1	0	0	1	0	0	1	0	1
55587351	1	1		50	1	1	2	0	0	1.73	89	1	1	0	0	1	0	0	1	0	0	0	1	1	0	0	1	0	0	0	0	1
55789533	3	2	2	62	2	0	2	0	80	1.6	90	2	1	0	0	1	0	0	0	0	0	0	1	0	0	0	1	0	0	0	0	1
55384334	1	1	2	69	2	1	1	0	40	1.73	85	1	1	1	1	0	0	0	0	0	0	0	1	1	0	0	1	0	1	0	0	1
55784026	3	2	2	37	1	1	1	0	0	1.72	91	2	1	0	0	0	0	1	0	0	0	1	0	1	1	1	1	0	1	0	0	1
55790032	3	2		50	2	1	2	0	0	1.81	78	1	1	0	0	1	0	0	0	0	0	1	0	0	1	1	1	0	0	1	0	1
2835602B	1	1	2	49	1	1	2	0	0	1.68	121	2	1	0	0	0	1	1	0	0	0	0	1	1	1	1	1	0	0	1	0	1
55770226	3	2	1	75	2	1	1	0	0	1.63	72	1	1	1	1	0	0	0	1	0	0	1	0	1	0	0	1	0	1	0	0	1
55783548	3	2	2	47	1	1	2	0	1	1.76	106	2	1	1	0	0	0	0	0	0	0	0	1	0	1	0	1	0	0	0	0	1
55789369	3	2	1	63	1	0	2	0	10	1.75	80	1	0	0	0	1	0	0	0	0	0	0	1	0	0	0	1	0	0	0	0	1
55785302	2	1	2	62	1	0	2	0	30	1.6	90	2	1	0	0	0	0	0	0	0	0	0	1	0	0	0	1	0	0	1	0	1
55780493	3	2	2	56	1	1	2	0	20	1.76	80	1	0	1	1	0	0	0	0	0	0	0	0	1	1	0	1	0	1	0	0	1
55791505	1	1		69	2	1	2	0	0	1.9	75	1	1	1	1	0	0	0	1	0	0	0	1	1	0	0	0	0	1	0	0	1
55744562	1	1	2	64	1	1	2	0	40	1.74	67	1	1	0	1	0	0	0	0	0	0	0	1	1	1	1	1	0	0	0	0	1
55542539	3	2	2	51	1	1	2	0	0	1.72	77	1	0	0	0	0	0	0	0	0	0	1	0	0	1	0	1	0	0	0	1	1
90234540	3	2	2	53	1	1	2	0	20	1.78	85	1	1	1	0	0	0	0	0	0	0	1	0	1	0	0	1	0	1	0	0	1
55779955	3	2	2	48	1	1	2	0	0	1.85	105	2	1	0	1	0	0	0	0	0	0	0	1	1	1	1	1	1	0	0	0	1
55789662	4	2	2	42	2	0	1	0	0	1.79	70	1	0	1	0	0	0	1	0	0	0	0	0	1	1	1	0	0	1	0	1	1
55746164	2	1	2	50	1	1	1	0	13	1.75	84	1	0	0	0	0	2	1	0	0	0	1	0	1	0	0	1	0	1	1	0	1
55589616	2	1	2	63	1	1	1	0	0	1.7	95	2	1	1	0	0	2	1	0	0	0	1	0	1	1	1	1	1	1	0	0	1
55776882	3	2		69	2	0	1	0	20	1.71	73	1	0	0	0	0	0	1	0	0	0	1	0	1	0	0	1	0	1	0	0	1
55783641	1	1	1	66	2	0	2	0	0	1.5	68	2	1	0	0	0	0	0	0	0	0	1	0	1	1	0	1	0	1	0	0	1
55397926	3	2	2	65	2	0	2	0	0	1.7	71	1	1	1	1	1	1	0	0	0	0	0	1	1	0	0	1	0	0	1	0	1
88240238	3	2	2	44	2	0	1	0	20	1.65	72	1	0	1	0	0	0	1	0	0	0	1	0	1	0	0	1	0	1	0	0	1
55782692	2	1		56	1	1	1	0	0	1.73	100	2	1	0	0	1	0	1	0	0	0	1	0	1	0	0	1	1	0	1	1	1
55775827	1	1	1	59	1	1	2	1	49,5	1.8	120	2	1	0	1	0	0	0	0	0	0	1	0	1	0	0	1	0	0	0	1	1
55787774	1	1	2	66	1	1	2	0	0	1.72	80	1	0	0	0	1	0	0	0	0	0	1	0	1	0	0	1	0	0	0	0	1
55599827	3	2	1	76	2	1	1	0	60	1.78	82	1	0	0	0	1	0	1	0	0	0	1	0	1	0	0	1	0	1	1	0	1
14214493	3	2	2	57	1	1	1	0	0	1.77	70	1	1	0	0	1	0	1	0	0	0	1	0	1	1	1	1	1	0	1	1	1
55595747	2	1	2	55	2	1	1	0	15	1.75	84	1	0	0	0	0	0	1	0	0	0	1	0	1	0	0	1	0	1	0	0	0
55582401	1	1	1	56	2	1	1	0	0	1.9	100	1	1	0	0	0	0	1	0	0	0	0	1	1	1	1	1	0	1	0	0	0
55610647	1	1	1	61	1	1	2	0	15	1.7	93	2	1	1	0	0	0	0	0	0	0	1	0	1	0	0	1	0	1	0	0	1
55586423	3	2	2	64	2	1	1	0	25	1.83	80	1	1	0	0	0	0	1	1	0	0	0	0	1	0	1	1	0	1	0	0	1
55780736	2	1	1	64	1	1	2	0	0	1.8	87	1	1	0	0	1	0	0	0	0	0	1	0	1	0	0	1	1	0	1	0	1

hr	nyha	nyha_dic	scholarity	age	lvef	gender	etiol	smok	packyears	height	weight	bmi	hipert	diabete	yslipidemi	atrialfyb	crt	ami	renaldis	stroke	cancer	ACI	ARB	Statins	Other antil	Nitrates	Betablock	Antiarrhytl	Antiplatel	Anticoagul	Digitalis	Diuretics
55612119	3	2	2	62	1	1	1	0	6	1.63	70	1	0	1	0	0	0	1	1	0	0	1	0	1	0	0	1	0	1	0	0	1
13701232	2	1	2	64	1	1	1	0	90	1.65	79	1	0	0	1	0	0	1	0	0	0	0	1	1	0	0	1	0	1	0	0	1
55790931	2	1		68	2	1	1	0	0	1.68	88	2	1	0	1	0	0	0	0	0	0	1	0	1	0	1	1	1	1	0	0	1
55782703	1	1	1	61	1	1	2	0	0	1.7	85	1	1	0	0	0	0	0	0	0	0	1	0	0	1	1	1	0	0	0	0	1
55767433	3	2	1	69	2	0	1	0	0	1.59	49	1	1	0	0	1	1	0	0	1	0	1	0	1	0	0	1	0	0	1	1	1
55783080	3	2	2	46	2	0	1	0	0	1.7	88	2	1	1	0	0	0	1	0	0	0	0	1	0	0	0	1	0	1	0	0	1
55789845	2	1	2	45	1	1	2	0	42	1.8	87	1	0	0	0	0	0	0	0	0	0	1	0	0	0	0	1	0	0	0	0	1
3032151E	2	1	2	57	1	1	2	1	40	1.82	67	1	0	0	0	1	0	0	0	0	0	1	0	0	0	0	1	1	0	1	0	1
55775470	3	2	2	55	1	1	2	0	100	1.9	113	2	1	0	0	0	0	0	0	0	0	1	0	0	1	0	1	0	0	0	0	1
55786707	3	2	2	47	1	1	1	0	10	1.9	92	1	1	1	0	0	0	1	0	0	0	0	1	1	1	1	1	0	1	0	1	1
55755812	3	2	2	75	2	0	2	0	0	1.53	58	1	1	0	0	0	0	0	0	0	0	1	0	1	1	1	1	0	0	0	0	1
55774371	2	1	1	71	2	0	2	0	0	1.52	68	1	1	1	1	0	0	1	0	0	1	0	1	1	0	0	1	0	0	0	0	1
55601087	2	1	1	65	1	0	2	0	0	1.66	65	1	1	1	0	0	0	0	0	0	0	1	0	0	1	1	1	0	0	1	0	1
3101067E	3	2	2	53	1	0	2	0	0	1.68	62	1	0	0	0	0	0	0	0	0	0	1	0	0	0	0	1	0	0	0	0	1
55777332	3	2	2	49	2	0	2	0	25	1.5	63	1	1	1	0	0	1	0	0	0	0	1	0	1	0	0	1	0	0	0	1	1
33569184	3	2	1	69	1	0	2	0	118	1.6	65	1	0	1	0	0	0	0	0	0	0	1	0	0	0	0	1	0	0	0	0	0
55764026	1	1	2	64	1	0	2	0	10	1.43	43	1	0	0	0	0	0	0	0	0	0	0	1	0	0	0	1	0	0	0	0	1
55569349	3	2	1	68	2	0	1	0	0	1.7	73.5	1	1	1	1	1	0	1	0	0	0	0	1	1	0	0	1	0	1	0	0	1
55788219	2	1	2	51	1	0	2	0	0	1.57	64	1	1	0	0	0	0	0	0	0	0	0	1	0	1	1	1	0	1	0	1	1
5142060K	3	2	2	64	2	0	2	0	0	1.7	80	1	0	0	0	0	1	0	0	0	0	0	1	0	0	0	1	0	0	1	1	1
55764014	3	2	1	63	1	0	2	0	0	1.55	64	1	1	1	1	0	0	0	0	0	0	0	1	1	1	1	1	0	1	0	0	1
55604288	3	2	1	73	2	0	1	0	0	1.58	54	1	1	0	0	0	0	1	0	0	0	1	0	1	0	0	1	0	1	0	0	1
15128349	1	1	2	68	2	1	1	1	25	1.75	66	1	1	1	0	0	0	1	0	0	0	1	0	1	0	0	1	0	1	0	0	0
55443214	4	2	2	66	2	0	1	0	0	1.6	60	1	1	1	1	0	0	1	1	0	0	0	1	1	1	0	1	0	1	0	0	1
55788727	3	2	2	45	1	1	1	0	6	1.8	94	1	1	0	0	1	0	1	0	0	0	1	0	1	0	0	1	1	0	1	1	1
55787575	1	1	2	56	2	1	1	0	0	1.7	92	2	0	1	0	0	0	1	0	0	0	1	0	1	0	0	1	0	1	0	0	1
55748411	1	1	1	50	1	1	2	0	0	1.8	100	2	1	1	0	0	0	0	0	0	0	1	0	1	0	0	1	0	1	0	0	1
5295663I	3	2	1	53	1	1	2	0	12	1.65	60	1	0	0	0	1	0	0	0	0	0	1	0	1	0	0	1	0	0	1	1	1
55788277	3	2	2	63	2	0	1	1	46	1.65	70	1	1	0	1	0	0	1	0	0	0	1	0	1	0	0	1	0	1	0	0	1
55606634	3	2		66	2	0	2	0	0	1.77	79	1	1	0	0	0	0	0	0	0	0	0	0	1	1	0	1	0	0	0	0	1
55768059	3	2	2	66	1	1	2	0	35	1.77	98	2	1	0	1	1	0	0	0	0	0	1	0	1	0	0	1	0	0	1	0	1
2651794B	3	2	2	68	2	1	2	0	0	1.65	46	1	0	0	0	1	2	0	0	0	0	1	0	0	1	1	1	1	0	0	0	1
55748108	3	2	2	64	1	1	2	0	60	1.75	95	2	1	1	1	0	0	0	0	0	0	1	0	0	0	0	1	0	1	0	0	1
55791784	2	1	2	67	1	1	2	0	0	1.77	68	1	1	0	0	0	0	0	0	0	0	0	1	1	0	0	1	0	1	0	0	1
55785679	3	2	2	53	1	1	2	0	0	1.6	65	1	0	0	0	0	0	0	0	0	0	1	0	0	0	0	1	0	0	0	0	1
55779636	2	1	1	61	1	1	2	0	2,5	1.78	80	1	0	0	0	0	0	0	0	0	0	1	0	1	0	0	1	0	0	0	1	0
55776576	3	2	2	38	1	0	1	0	7,5	1.75	72	1	1	0	1	0	0	1	0	0	0	1	0	1	1	1	1	0	1	0	0	1
55784190	3	2	2	46	1	1	2	0	0	1.7	75	1	0	0	0	0	0	0	1	0	0	1	0	0	0	0	1	0	0	0	0	1
55592064	2	1	2	39	1	0	2	0	0	1.5	60	1	1	0	1	0	2	0	0	0	0	1	0	0	0	0	1	1	0	0	0	1
55776342	2	1		36	2	0	1	0	0	1.87	80	1	1	0	0	0	0	1	0	0	0	1	0	0	0	0	1	0	0	1	0	1
2949097E	3	2	2	78	1	0	2	0	0	1.45	50	1	1	1	1	0	1	0	0	0	0	0	1	1	1	0	1	0	1	0	1	1
55772440	2	1	2	30	2	1	2	0	0	1.63	53	1	0	0	0	0	0	0	0	0	0	1	0	0	0	0	1	0	0	0	0	0
2955647J	3	2	1	56	2	1	2	0	0	1.84	73	1	0	0	0	1	2	0	0	0	0	0	1	0	0	0	1	1	0	1	0	1
55583776	2	1	2	58	2	1	1	0	0	1.7	75	1	0	0	0	0	0	1	0	0	0	0	1	0	0	0	0	0	0	0	0	1
55790131	3	2	2	75	1	1	1	0	0	1.85	110	2	1	1	0	0	0	0	0	0	0	0	1	1	1	1	1	0	1	1	0	1
55786416	1	1	2	52	1	1	1	0	0	1.87	102	1	0	1	1	1	0	1	0	0	0	1	0	0	1	0	1	0	0	1	1	1
5252340C	1	1	2	52	1	1	2	1	3,75	1.78	98	2	0	1	0	1	0	0	0	0	0	1	0	0	0	0	1	0	0	1	1	1
13981413	3	2	2	68	2	0	1	0	0	1.6	70	1	1	1	1	0	0	0	0	0	0	0	1	0	0	0	1	0	1	0	0	1
55779073	3	2	2	65	1	1	2	0	60	1.7	117	2	1	0	0	1	0	0	0	0	0	1	0	0	0	0	1	0	0	1	0	1
55464972	2	1	2	70	1	1	1	0	0	1.69	87	2	1	0	1	0	0	1	1	0	0	1	0	1	1	1	1	0	1	0	0	1
55779649	3	2	2	63	1	1	2	0	0	1.55	87.5	2	1	1	1	1	0	0	0	0	0	1	0	1	1	1	1	1	0	1	0	1
55787210	3	2	2	63	1	1	2	0	0	1.7	80	1	1	0	1	0	0	0	0	0	0	0	1	1	1	0	1	0	1	0	0	1
90358770	1	1	2	59	2	0	2	0	0	1.62	58	1	1	1	0	0	0	0	0	0	0	1	0	1	0	0	1	0	0	0	0	1
55771193	3	2	2	40	1	0	2	0	3	1.53	102	2	1	0	0	0	0	0	0	0	0	0	1	0	0	0	1	0	0	0	0	1
55522343	3	2	2	58	1	1	1	0	0	1.7	70	1	1	0	0	0	2	0	0	0	0	0	0	1	1	0	1	0	1	0	1	1
55780092	2	1	2	74	2	1	2	0	0	1.68	85	2	1	0	0	1	2	0	0	0	0	1	0	0	0	0	1	0	0	1	0	0
55609981	2	1	2	55	1	1	2	0	0	1.82	118	2	1	0	0	1	0	0	0	0	0	1	0	1	0	0	1	1	0	1	0	1
55377777	2	1	2	74	1	0	2	0	0	1.56	56	1	1	0	1	0	0	0	0	0	0	1	0	1	0	0	1	0	0	0	0	1
55605513	3	2	1	60	2	1	1	0	116	1.54	70	1	0	0	0	0	0	1	0	0	0	1	0	1	0	0	1	0	1	0	0	1
55789281	2	1	1	61	1	0	1	0	0	1.6	75	1	1	0	1	0	0	1	0	1	0	1	0	1	0	0	1	0	0	0	0	1

k_1a	k_1b	k_1c	k_physlim	k_2	k_3	k_4	k_5	k_symp	k_6	k_7	k_qol	k_8a	k_8b	k_8c	k_soclim	k_tot	k_dic	k_quart	m_1	m_2	m_3	m_4	m_5	m_6	m_7	m_8	m_9	m_10	m_11	m_12	m_13	m_14	m_15	m_16	m_17	m_18	m_19	m_20	m_21	m_fis	m_emo	m_tot	m_dic	m_quart
3	3	1	33	5	4	7	5	88	4	3	63	3	3	5	67	63	2	3																										
3	2	2	33	1	2	1	5	29	1	2	13	2	2		25	25	1	2	5	5	5	0	0	0	0	5	5	5	5	5	4	0	0	0	5	5	5	5	4	24	24	68	2	3
1	1	1	0	4	1	7	5	69	1	1	0	1	1	1	0	17	1	1	2	5	5	5	5	0	5	5	5	5	2	3	5	5	3	2	3	4	5	4	5	35	21	83	2	4
4	3	2	50	5	2	6	5	75	2	2	25	2	2	2	25	44	1	2																										
5	3	4	75	5	5	7	5	92	4	3	63	5	2	2	50	70	2	3																										
3	2	2	33	4	2	2	4	46	2	2	25		1	3	25	32	1	2																										
4	2	1	33	2	2	4	1	23	3	1	25	1	3		25	27	1	2																										
4	5	1	58	5	2	7	5	79	1	3	25	1	1		0	41	1	2	0	0	5	4	3	0	5	5	5	5	3	2	4	0	5	0	0	3	4	0	4	23	11	57	2	3
5	5	5	100	5	7	7	5	100	5	5	100	5	5		100	100	2	4	0	0	0	0	0	0	0	0	0	0	0	0	0	0	0	0	0	0	0	0	0	0	0	0	1	1
2	3	1	25	1	1	1	5	25	1	1	0	2	1	1	8	15	1	1	5	5	5	5	5	4	5	5	5	5	5	5	5	0	0	0	5	5	5	4	4	44	23	87	2	4
5	5	5	100	5	7	7	5	100	5	5	100	5	5	5	100	100	2	4																										
3	3	2	42	5	4	6	5	83	2	1	13	2	2	2	25	41	1	2																										
2	3	1	25	3	4	4	3	50	2	2	25	3	2	2	33	33	1	2	5	5	5	5	5	5	5	5	5	5	5	5	5	5	5	3	5	5	5	2	2	45	19	97	2	4
5	3		75	4	5	7	5	85	5	3	75					78	2	4																										
5	5	3	83	3	7	7	5	88	5	3	75	3	3	5	67	78	2	4	3	0	5	0	0	0	0	5	0	0	0	3	3	0	0	0	0	0	3	3	0	14	6	25	1	1
5	3	2	58	3	5	5	5	71	4	3	63	4	4	5	83	69	2	3	0	0	4	4	3	0	0	5	4	2	4	4	3	0	4	0	3	2	4	3	4	18	16	53	2	3
5	5	5	100	5	6	6	5	92	5	4	88	4	3	5	75	89	2	4																										
3	1	1	17	5	2	2	2	40	2	2	25	3	2	1	25	27	1	2	5	5	5	5	5	4	5	5	5	5	5	5	5	0	0	0	5	5	5	5	5	44	25	89	2	4
2	2	1	17	5	1	1	2	31	2	2	25	3	2	1	25	24	1	1																										
5	3	2	58	3	4	3	5	58	3	3	50	3	3	4	58	56	2	3																										
5	4	3	75	4	5	4	5	73	4	3	63	4	3		63	68	2	3	2	3	2	1	0	5	5	5	2	2	5	2	3	0	5	5	3	2	5	4	5	23	19	66	2	3
2	2	2	25	5	1	4	2	44	2	1	13	2	2		25	27	1	2																										
3	2	1	25	3	2	2	2	27	1	1	0	1	2	1	8	15	1	1	3	4	4	4	5	4	5	5	5	5	4	4	4	4	5	5	5	5	5	5	5	37	25	95	2	4
4	2		50	1	3	3	1	17	2	1	13	2	2	2	25	26	1	2																										
4	2	2	42	5	4	3	3	58	3	3	50	3	3	5	67	54	2	3	0	3	5	3	4	3	0	0	5	4	3	4	2	0	0	3	0	1	5	0	3	24	9	48	1	2
5	4	1	58	5	7	7	5	100	5	5	100	5	5		100	90	2	4																										
2	1	1	8	1	1	1	1	0	1	1	0	1	1	1	0	2	1	1	5	5	5	5	5	5	5	5	5	5	5	5	5	0	5	5	0	3	5	5	5	45	18	93	2	4
5	5	5	100	5	7	7	5	100	5	4	88	5	5	5	100	97	2	4																										
3	3	1	33	3	2	2	5	46	4	3	63	3	1	3	33	44	1	2	3	5	5	5	3	4	0	5	5	4	3	5	5	0	5	3	4	5	5	4	5	35	23	83	2	4
5	5	1	67	4	1	3	1	27	2	2	25	2	1	1	8	32	1	2	0	0	3	0	0	0	0	0	0	5	0	2	0	0	0	0	0	0	3	0	0	5	3	13	1	1
5	3	2	58	5	5	5	5	83	5	3	75	3	3	3	50	67	2	3	0	0	4	4	4	0	0	5	5	5	0	0	0	0	0	0	0	0	0	4	0	12	4	31	1	2
5	5	4	92	5	7	7	5	100	5	5	100	5	5	5	100	98	2	4	0	0	0	0	0	0	0	0	1	0	1	0	1	0	2	1	0	0	0	0	0	1	0	6	1	1
3	1		25	5	2	7	5	79	2	1	13	2	1		13	32	1	2																										
5	5	4	92	5	5	5	5	83	4	3	63	3	3	2	42	70	2	3	0	3	3	0	0	0	0	0	3	5	0	3	3	0	0	0	0	0	0	0	3	12	3	23	1	1
5	2	1	42	2	3	4	2	33	2	1	13	2	2	3	33	30	1	2																										
5	2	2	50	2	1	1	1	6	1	1	0	1	1	1	0	14	1	1	5	5	5	5	5	5	5	5	5	5	5	5	5	5	5	5	5	5	5	5	5	45	25	105	2	4
5	3	3	67	5	6	6	5	92	3	3	50	3	3	3	50	65	2	3	0	0	1	1	1	0	0	2	0	2	1	0	1	0	1	1	1	0	1	0	0	4	2	13	1	1
3		2	38	2	4	3	3	40	5	3	75	2	2	2	25	44	1	2	3	5	4	5	4	3	3	5	3	4	5	1	3	0	0	3	0	1	2	0	0	31	3	54	2	3
3				1	5	1	5	42	2	1	13	1	1	6	42	32	1	2																										
4	2	1	33	5	6	7	5	96	2	2	25	3	1	2	25	45	1	2																										
3	2		38	4	4	6	5	77	3	1	25	1	2	2	17	39	1	2																										
2	3	2	33	5	2	2	2	40	3	5	75	2	3	3	42	47	1	2																										
3				5	7	7	5	100	5	5	100	5	5		100	100	2	4	0	0	0	0	0	0	0	0	0	0	0	0	0	0	0	0	0	0	0	0	0	0	0	0	1	1
3	5	3	67	3	5	7	3	67	3	2	38	3	3	4	58	57	2	3	4	3	2	3	4	3	3	4	3	3	3	4	3	2	4	2	4	4	4	1	1	29	14	64	2	3
3	3	3	50	5	5	5	3	71	3	3	50	3	2		38	52	2	3	0	2	4	3	4	5	4	3	2	4	4	3	2	4	5	3	0	2	4	3	4	27	13	65	2	3
3	2	2	33	4	2	2	1	27	1	3	25	1	1	1	0	21	1	1	5	5	5	5	4	5	4	5	5	5	5	5	5	0	3	3	3	4	5	3	5	43	20	89	2	4
2	2		25	2	2	2	1	15	2	2	25	2	1	2	17	20	1	1																										
2	1	1	8	1	1	1	1	0	1	1	0	1	1	1	0	2	1	1	5	5	5	5	5	5	5	5	5	5	5	5	5	5	5	5	5	5	5	3	5	45	23	103	2	4
5	2	1	42	5	3	4	5	71	2	1	13	1	1	4	25	38	1	2	0	0	5	3	3	5	3	4	5	5	5	4	4	0	0	0	0	0	3	0	0	27	3	49	1	2
3	3	2	42	5	5	5	5	83	3	3	50	3	3	5	67	60	2	3	3	3	4	3	0	3	0	0	3	0	3	3	3	0	0	3	0	0	3	2	0	22	5	36	1	2
3	2	1	25	5	2	1	3	42	3	3	50	3	2	3	42	40	1	2	3	4	5	5	4	5	3	5	3	5	5	5	5	0	4	0	3	2	5	5	5	39	20	81	2	4
5	3	2	58	1	7	5	5	67	5	5	100	5	5	5	100	81	2	4	0	0	5	0	0	0	0	0	0	5	0	0	0	0	5	5	0	5	5	5	5	5	20	40	1	2
2	2	1	17	2	2	4	5	48	1	1	0	1	2		13	19	1	1	5	5	5	4	4	5	4	5	5	5	2	5	5	0	4	2	2	5	4	3	3	42	17	82	2	4
4	3	2	50	2	3	2	3	31	2	3	38	2	1	3	25	36	1	2	4	5	5	4	4	4	3	3	4	3	3	3	4	0	0	0	4	5	5	4	5	36	23	72	2	3
3	3	1	33	3	4	4	4	56	3	2	38	2	1	1	8	34	1	2	2	1	4	4	3	2	4	5	4	5	3	4	5	0	0	0	0	3	4	2	5	29	14	60	2	3
5	5	4	92	5	7	7	5	100	5	4	88	5	5	5	100	95	2	4																										
5	5	3	83	5	7	7	5	100	5	5	100	5	5	4	92	94	2	4	0	5	5	5	5	5	5	5	5	5	5	5	5	0	0	0	0	0	0	5	0	40	5	65	2	3
5	2	1	42	5	2	2	3	46	1	1	0	1	1	2	8	24	1	1	5	5	4	3	5	5	5	5	5	5	4	5	5	5	5	3	5	5	5	3	5	42	23	97	2	4
5	5	4	92	5	4	4	5	75	3	3	50	3	3	5	67	71	2	3	1	2	3	3	2	2	0	4	3	3	2	1	4	0	4	1	1	3	4	3	0	18	11	46	1	2
5	3	2	58	5	5	7	5	92	3	3	50	3	3	5	67	67	2	3																										
5	5	3	83	4	3	4	5	65	3	2	38	3	3	2	42	57	2	3	0	4	2	3	2	4	2	1	3	2	1	3	3	1	2	3	1	3	3	1	2	23	10	46	1	2
4	2	1	33	5	2	4	5	67	2	1	13	3	1		25	34	1	2	0	0	3	0	3	3	3	5	0	2	5	0	4	0	2	0	0	3	0	3	0	16	6	36	1	2
3	2	2	33	3	1	1	5	38	3	3	50		2			40	1	2	1	3	5	4	4	5	3	5	5	5	5	5	5	0	5	0	0		5	5	4	35	14	74	2	3
5	3	2	58	5	7	7	5	100	4	5	88		4	5	88	83	2	4	0	0	5	0	0	0	0	0	5	5	5	3	3	0	5	0	5	0	0	3	0	11	8	39	1	2
3	4	3	58	5	7	3	5	83	2	3	38	3	5	5	83	66	2	3	0	0	5	2	0	0	0	5	5	5	5	2	5	0	5	4	0	2	3	2	0	14	7	50	1	2
5	3	2	58	5	2	4	5	67	2	1	13	2	3		38	44	1	2																										
5	4	2	67	5	3	7	5	83	5	5	100		3			83	2	4																										
5	3	3	67	5	4	5	1	54	4	1	38	5	3	3	67	56	2	3	0	0	0	0	0	3	3	3	3	3	3	0	5	0	3	0	0	0	3	0	0	11	3	29	1	2
4	3	1	42	4	4	3	5	65	3	2	38	3	2	3	42	46	1	2	0	3	5	4	2	1	0	0	3	0	4	1	5	0	3	1	0	1	1	5	0	21	7	39	1	2
2	3	1	25	5	3	6	5	79	3	3	50	2	1	0	13	42	1	2																										
5	3	3	67	3	7	7	5	88	5	4	88	5	5	5	100	85	2	4																										
3	2	3	42	2	5	5	5	65	3	3	50	3	2	5	58	54	2	3																										
3	2	1	25	2	1	1	1	6	1	1	0	1	1	1	0	8	1	1	5	5	5	5	3	3	0	5	5	5	3	5	5	0	3	0	0	3	5	3	5	36	16	73	2	3
5	3	2	58	2	2	4	1	23	2	1	13	2	2		25	30	1	2	3	5	3	3	3	5	3	5	5	3	5	3	5	3	5	0	5	5	5	3	0	33	18	77	2	3
2	2	2	25	5	1	2	3	42	4	5	88	4	2	4	58	53	2	3	0	5	5	5	5	5	0	5	5	0	0	5	5	0	5	5	5	5	0	0	0	35	10	65	2	3
5	3	1	50	5	5	7	5	92	3	5	75	3	3		50	67	2	3																										
5	5		100	5	7	7	5	100	5	3	75	5	3	4	75	88	2	4	0	0	0	5	0	0	0	5	0	0	0	0	0	0	0	0	0		0	0	0	5	0	10	1	1
3	1	1	17	3	1	4	1	25	1	1	0	2	1	2	17	15	1	1	0	5	5	5	5	5	5	5	5	5	5	5	5	5	5	5	5	5	5	5	5	40	25	100	2	4
3	3	2	42	3	4	4	4	56	3	3	50	3	3	3	50	49	1	2	5	5	5	5	3	5	2	5	4	4	2	3	5	0	0	0	0	3	5	4	4	38	16	69	2	3

k_1a	k_1b	k_1c	k_physlim	k_2	k_3	k_4	k_5	k_symp	k_6	k_7	k_qol	k_8a	k_8b	k_8c	k_soclim	k_tot	k_dic	k_quart	m_1	m_2	m_3	m_4	m_5	m_6	m_7	m_8	m_9	m_10	m_11	m_12	m_13	m_14	m_15	m_16	m_17	m_18	m_19	m_20	m_21	m_fis	m_emo	m_tot	m_dic	m_quart
5	3	2	58	5	5	6	5	88	4	3	63	4	4		75	71	2	3	1	0	5	0	0	0	0	0	0	0	5	0	5	0	0	0	0	0	5	0	5	11	10	26	1	1
2	4	2	42	5	5	7	3	79	3	3	50	3	3	4	58	57	2	3	0	3	2	0	2	4	3	4	2	3	0	5	2	0	0	2	0	0	2	2	0	21	4	36	1	2
3	3	1	33	5	3	2	5	63	3	2	38	3	2	2	33	42	1	2	0	5	5	4	5	5	0	5	5	5	5	3	5	0	0	5	0	0	5	5	5	32	15	72	2	3
3	4	1	42	5	2	5	3	58	3	3	50	4	2	4	58	52	2	3																										
5	3	1	50	3	4	4	5	63	3	3	50	3	3	3	50	53	2	3	4	5	4	4	4	3	0	3	3	3	0	0	3	4	0	0	0	0	3	4	0	27	7	47	1	2
4	3	1	42	5	4	4	1	50	3	3	50		3			47	1	2	0	5	5	4	3	4	0	0	3	0	2	3	4	0	2	0	0	0	4	5	4	28	13	48	1	2
3	3	2	42	5	5	5	5	83	3	3	50	3	4	5	75	63	2	3	0	0	3	3	3	3	0	4	3	3	0	5	5	0	0	0	0	0	0	0	0	22	0	32	1	2
5	4		88	5	5	7	5	92	4	4	75	5	4	4	83	84	2	4	0	0	2	0	0	0	0	0	0	1	0	0	3	0	5	0	0	0	2	1	2	5	5	16	1	1
1	1	1	0	5	2	2	1	33	2	2	25	1	1	1	0	15	1	1																										
2	2	1	17	2	4	1	2	25	1	1	0	1	1	2	8	13	1	1	5	5	5	2	3	5	4	5	5	5	3	5	5	4	3	3	3	3	5	2	3	39	16	83	2	4
4	4	2	58	5	4	5	5	79	4	3	63	2	2	1	17	54	2	3	0	1	2	2	2	1	1	5	2	5	5	2	2	0	4	5	5	5	5	3	2	13	20	59	2	3
5	3	3	67	2	5	5	3	52	4	3	63	2	4	4	58	60	2	3	1	3	3	2	3	3	3	2	2	3	3	2	3	3	2	3	3	2	3	3	3	23	14	55	2	3
3	3	1	33	4	1	1	1	19	2	2	25	2	3	3	42	30	1	2	2	5	5	4	4	5	4	5	5	5	4	4	5	1	4	5	5	4	5	5	4	38	23	90	2	4
3	2	2	33	3	2	3	5	50	3	3	50	2	2	2	25	40	1	2																										
5	3	2	58	5	2	2	5	58	5	3	75	5	3		75	67	2	3	0	0	5	5	5	4	0	4	5	4	0	3	5	0	5	0	0	0	5	0	0	27	5	50	1	2
3	5	3	67	2	2	2	4	33	3	2	38	2	3	2	33	43	1	2																										
2	3	2	33	5	3	3	4	60	4	3	62,5	4	5	5	92	62	2	3	0	3	2	4	0	3	3	0	0	2	2	1	3	0	1	0	0	3	5	3	3	19	14	38	1	2
1	2	1	8	2	4	3	4	46	2	1	13	2	2	2	25	23	1	1																										
3	3	2	42	2	4	7	5	69	3	5	75	3	3	5	67	63	2	3	0	5	5	2	3	0	0	5	5	5	2	3	3	0	4	3	0	0	5	2	1	21	8	53	2	3
3	3	1	33	5	4	3	5	71	1	2	13	1	1	3	17	33	1	2	0	0	5	5	5	0	5	5	5	5	5	5	5	0	2	3	2	4	5	2	2	30	15	70	2	3
5	3	3	67	5	5	5	5	83	5	4	88	5	3	5	83	80	2	4																										
3	1	1	17	3	2	2	1	21	1	2	13	1	1	2	8	15	1	1	5	5	5	5	5	5	5	5	5	5	5	5	5	0	5	5	0	5	5	5	5	45	20	95	2	4
2	2	1	17	3	1	1	1	13	1	1	0	1	1	1	0	7	1	1	4	5	5	5	5	4	5	5	5	5	5	4	5	0	3	4	5	4	5	4	5	42	23	92	2	4
5	3	3	67	5	6	5	5	88	3	4	63	3	1	5	50	67	2	3																										
4	2	1	33	5	6	5	4	81	3	2	38	3	2	4	50	51	2	3	2	0	3	4	3	1	0	5	3	1	5	4	4	0	0	0	1	3	5	4	2	21	15	50	1	2
3	3	1	33	5	3	7	5	83	5	3	75	4	4	2	58	63	2	3	0	0	5	0	3	4	4	5	5	5	4	0	4	0	5	5	3	4	5	3	5	20	20	69	2	3
5	2	2	50	5	4	4	4	69	3	3	50	3	3	3	50	55	2	3																										
3	2	1	25	2	1	1	1	6	1	1	0	1	1	2	8	10	1	1	5	5	5	5	5	5	0	5	5	0	0	5	5	3	5	3	2	5	1	3	2	40	13	74	2	3
5	2	3	58	4	4	5	5	73	3	4	63	3	3	5	67	65	2	3																										
3	3	3	50	3	5	5	5	71	3	3	50	1	3	2	25	49	1	2	3	0	4	3	4	2	5	5	0	0	0	0	3	0	5	0	5	5	0	0	0	24	10	44	1	2
4	4	3	67	5	7	7	5	100	5	4	88	5	5		100	89	2	4																										
5	4	2	67	5	2	6	5	75	3	3	50	3	3		50	60	2	3	0	5	5	3	0	0	0	4	4	3	0	2	3	0	5	0	3	3	5	2	4	18	17	51	1	2
2	2	2	25	5	1	5	1	42	2	1	13	2	2	2	25	26	1	2																										
2	2	2	25	5	3	6	5	79	2	2	25	1	2	1	8	34	1	2																										
3	3	2	42	5	3	4	5	71	4	3	63	3	2	3	42	54	2	3																										
3	2	2	33	2	1	4	1	19	1	2	13	3	4	4	67	33	1	2	5	4	3	5	5	4	3	5	5	5	5	5	5	5	5	4	5	4	5	5	5	39	24	97	2	4
2	1	1	8	1	1	2	1	4	1	1	0	1	1	1	0	3	1	1	3	3	5	5	4	4	3	5	5	5	4	4	4	0	1	1	1	0	2	2	2	35	7	63	2	3
5	5	3	83	2	7	7	5	81	5	3	75	3	3	5	67	77	2	4	2	0	3	0	2	0	0	0	2	0	4	0	2	0	5	4	0	2	0	0	0	9	2	26	1	1
2	3	1	25	2	2	5	1	27	2	1	13	2	2		25	22	1	1	5	4	5	3	5	5	3	5	5	5	5	3	5	0	5	5	5	5	5	5	5	38	25	93	2	4
2	1	1	8	5	2	2	1	33	2	1	13	1	1		0	14	1	1																										
3	3	2	42	5	5	6	5	88	5	5	100	3	2	4	50	70	2	3																										
5	5	5	100	5	7	7	5	100	5	3	75	5	5	5	100	94	2	4	0	0	0	0	0	0	0	0	0	0	5	0	0	0	2	0	0	0	0	0	0	0	0	7	1	1
5	3	1	50	5	7	6	5	96	4	3	63	2				69	2	3	0	5	5	4	4	5	4	3	5	0	5	4	5	0	0	0	0	0	0	0	5	36	5	54	2	3
5	4	3	75	5	1	6	1	46	4	1	38	4	4	4	75	58	2	3	5	5	5	5	5	5	5	5	5	5	5	5	5	0	5	5	5	5	5	5	5	45	25	100	2	4
5	5	1	67	3	5	7	5	79	5	5	100	5	5	1	67	78	2	4																										

Table S2. Available Data 2

Subtitle						
	data not collected or missing					
sex:	0 female	hr: hospital record	k_1a	question 1a of KCCQ-12	m_1	question 1 of MLHFQ
	1 male	nyha_dic: nyha dicotomizado	k_1b	question 1b of KCCQ-12	m_2	question 2 of MLHFQ
		nyha: New York Heart Association	k_1c	question 1c of KCCQ-12	m_3	question 3 of MLHFQ
etiol	1 ischemic	bmi: body index mass	k_physlim	Score of the domain - Physical Limitation	m_4	question 4 of MLHFQ
	2 non-ischemic	hipert: hipertensão arterial sistêmica	k_2	question 2 of KCCQ-12	m_5	question 5 of MLHFQ
		diabete: diabetes mellitus	k_3	question 3 of KCCQ-12	m_6	question 6 of MLHFQ
smok	0 non-smoker or ex-smoker	atrialfyb: atrial fibrillation	k_4	question 4 of KCCQ-12	m_7	question 7 of MLHFQ
	1 active smoker	ami: ainfarto agudo do miocardio	k_5	question 5 of KCCQ-12	m_8	question 8 of MLHFQ
		renaldis: doença renal crônica	k_symp	Score of the domain - Symptoms	m_9	question 9 of MLHFQ
comorbidities	0 no	KCCQ - 12: kansas city cardiomyopathy questionnaire-12	k_6	question 6 of KCCQ-12	m_10	question 10 of MLHFQ
	1 yes	MLHFQ: minnesota living heart failure questionnaire	k_7	question 7 of KCCQ-12	m_11	question 11 of MLHFQ
		ACI: Angiotensin-converting enzyme inhibitors	k_qol	Score of the domain - Quality of Life	m_12	question 12 of MLHFQ
crt	0 absence of device	ARB: Angiotensin receptor blockers	k_8a	question 8a of KCCQ-12	m_13	question 13 of MLHFQ
	1 pacemaker	Other antihyp: Other antihypertensive drugs	k_8b	question 8b of KCCQ-12	m_14	question 14 of MLHFQ
	2 implantable cardioverter-defibrillator	QoL: Quality of Life	k_8c	question 8c of KCCQ-12	m_15	question 15 of MLHFQ
			k_soclim	Score of the domain - Social Limitation	m_16	question 16 of MLHFQ
nyha_ajt	1 classes I and II		k_tot	escore total do KCCQ-12	m_17	question 17 of MLHFQ
	2 classes III and IV		k_dic	Overall score of KCCQ-12 dichotomized	m_18	question 18 of MLHFQ
			k_quart	Overall score of KCCQ-12 divided into quartiles	m_19	question 19 of MLHFQ
lvef	1 LVEF ≤ 30%				m_20	question 20 of MLHFQ
	2 LVEF >30%				m_21	question 21 of MLHFQ
					m_tot	Overall score of MLHFQ
bmi	1 < 30.0kg/m2				m_dic	Overall score of MLHFQ dichotomized
	2 ≥ 30.0kg/m2				m_quart	Overall score of MLHFQ divided into quartiles
					m_fis	MLHFQ Physical Domain
Scholarity:	1 Illiterate or elementary school ≤ 4 years				m_emo	MLHFQ Emotional Domain
	2 Incomplete elementary school > 4 years or higher level of education					
k_dic	1 ≤ 49 pointos (very poor to fair quality of life)					
	2 ≥ 50 pointos (fair to excellent quality of life)					
k_quart	1 (0-24 pontos -Very poor to poor QoL)					
	2 (25-49 - Very poor to fair QoL)					
	3 (50-74 - Fair to good QoL)					
	4 (75 - 100 - Good to excellent QoL)					
m_dic	1 ≤ 52 pontos (Fair to excellent QoL)					
	2 ≥ 53 pontos (Very poor to fair QoL)					
m_quart	1 (0-26 pontos - Good to excellent QoL)					
	2 (27-52 - Fair to good QoL)					
	3 (53-78 - Fair to poor QoL)					
	4 (79-105 - Poor to very poor QoL)					
